# Investigation of the role of miRNA variants in neurodegenerative brain diseases

**DOI:** 10.3389/fgene.2025.1506169

**Published:** 2025-02-26

**Authors:** Alexandros Frydas, Rita Cacace, Julie van der Zee, Christine Van Broeckhoven, Eline Wauters

**Affiliations:** ^1^ VIB Center for Molecular Neurology, Antwerp, Belgium; ^2^ Department of Biomedical Sciences, University of Antwerp, Antwerp, Belgium

**Keywords:** frontotemporal dementia, Alzheimer’s disease, noncoding RNA, miRNAs, rare genetic variants

## Abstract

**Introduction:**

miRNAs are small noncoding elements known to regulate different molecular processes, including developmental and executive functions in the brain. Dysregulation of miRNAs could contribute to brain neurodegeneration, as suggested by miRNA profiling studies of individuals suffering from neurodegenerative brain diseases (NBDs). Here, we report rare miRNA variants in patients with Alzheimer’s dementia (AD) and frontotemporal dementia (FTD).

**Methods:**

We initially used whole exome sequencing data in a subset of FTD patients (n = 209) from Flanders-Belgium. We then performed targeted resequencing of variant-harboring miRNAs in an additional subset of FTD patients (n = 126) and control individuals (n = 426). Lastly, we sequenced the *MIR885* locus in a Flanders-Belgian AD cohort (n = 947) and a total number of n = 755 controls.

**Results:**

WES identified rare seed variants in *MIR656, MIR423, MIR122 and MIR885* in FTD patients. Most of these miRNAs bind to FTD-associated genes, implicated in different biological pathways. Additionally, some miRNA variants create novel binding sites for genes associated with FTD. Sequencing of the *MIR885* locus in the AD cohort initially showed a significant enrichment of *MIR885* variants in AD patients compared to controls (SKAT-O, p-value = 0.026). Genetic association was not maintained when we included sex and *APOE* status as covariates. Using the miRVaS prediction tool, variants rs897551430 and rs993255773 appeared to evoke significant structural changes in the primary miRNA. These variants are also predicted to strongly downregulate mature *miR885* levels, in line with what is reported for *MIR885* in the context of AD.

**Discussion:**

Functional investigation of miRNAs/variants described in this study could propose novel miRNA-mediated molecular cascades in FTD and AD pathogenicity. Furthermore, we believe that the genetic evidence presented here suggests a role for *MIR885* in molecular mechanisms involved in AD and warrants genetic follow-up in larger cohorts to explore this hypothesis.

## 1 Introduction

MicroRNAs (miRNAs) are small (≈18–22 nt) noncoding single-stranded RNA molecules. They predominantly act by binding to the 3′ untranslated regions (UTRs) of complementary mRNA targets, leading to reduced target expression. Most are ubiquitously expressed in mammals, while others display tissue-specific enrichment, suggesting distinct functions in these tissues (Landgraf et al., 2007; Ludwig et al., 2016). After their transcription, miRNAs are subjected to two cleavage steps. The first takes place in the nucleus, where the complex consisting of the DGCR8 (Di George syndrome critical region gene 8) and the Drosha ribonuclease processes the primary miRNA (pri-miRNA) to the precursor miRNA (pre-miRNA). Following transport to the cytoplasm by Exportin 5′ and the Ran-GTP factor, RNA III enzyme Dicer cuts off the terminal loop to generate the mature miRNA duplex. Ultimately, one of the strands (3p and 5p miRNAs isoforms) is loaded onto the Argonaute protein of the RNA-induced silencing complex to guide it to its target mRNAs (Cammaerts et al., 2015b; Sadlon et al., 2019; Zeng et al., 2005).

The involvement of miRNAs in neurodegenerative processes has become more evident following increased research focus on the noncoding part of the genome. Expression profiling in the human brain has shown dysregulated miRNAs in neurodegenerative phenotypes, like Alzheimer’s disease (AD) and Parkinson’s disease (PD) (Schulz et al., 2019; Takousis et al., 2019). Similar studies in serum and plasma also showcase the use of miRNAs as diagnostic biomarkers for neurodegenerative brain diseases (NBDs) (Sheinerman et al., 2017), including frontotemporal dementia (FTD) (Grasso et al., 2019) and amyotrophic lateral sclerosis (ALS) (Freischmidt et al., 2015).

Genetic variation can impact miRNA function at different levels. For instance, mutations in 3′ UTRs can create or distort existing miRNA binding sites, leading to differential mRNA expression of the target gene. Such cases have been described for NBD-associated genes, such as α-synuclein in PD (Junn et al., 2009; Su et al., 2018) and progranulin (*GRN*) in FTD (Rademakers et al., 2008). Alternatively, genetic variants within miRNA genes can modulate their functions in different ways, for example, by affecting the processing during maturation or by altering the “seed” sequence with which the miRNA binds to its complementary mRNA target (Cammaerts et al., 2015a). Accordingly, meta-analyses of GWAS performed on AD and PD patients identified miRNA variants associated with disease pathogenesis (Ghanbari et al., 2016a; Ghanbari et al., 2016b).

In the present study, we are investigating the implication of miRNA variants in FTD. Based on a list of brain-expressed miRNAs (Cammaerts et al., 2015b), we are looking for miRNA variants in FTD patients with available whole exome sequencing (WES) data. We believe that our approach, focusing exclusively on variants in noncoding molecules like miRNAs, could improve our understanding of the genetic etiology of FTD, as such variation is regularly overlooked in most GWAS.

## 2 Methods

### 2.1 Study cohorts

#### 2.1.1 FTD cohort

FTD patients were sampled by members of the Belgian Neurology (BELNEU) Consortium as part of an ongoing multicenter collaborative study of neurology departments and memory clinics across Flanders-Belgium. We selected 335 unrelated FTD patients with well-documented clinical presentation (mean age at onset (AAO): 62.9 ± 10.3, range: 29–85, 47.9% female). 15.6% of FTD patients carried a known pathogenic mutation in a causal gene for frontotemporal lobar degeneration (*C9orf72, GRN, MAPT, TBK1, VCP,* or *CHMP2B*). Clinical diagnosis of FTD was made in accordance with international consensus criteria (Gorno-Tempini et al., 2011; Rascovsky et al., 2011).

#### 2.1.2 AD cohort

The AD cohort consisted of unrelated individuals recruited from neurology centers at university and general hospitals of the Flanders-Belgian region. Overall, we included 685 late-onset AD (LOAD) individuals (mean AAO: 77.9 ± 5.8, range: 66–99, 66.7% female) and 262 early-onset AD (EOAD) individuals (mean AAO: 59.1 ± 5.4, range 37–65, 56.4% female). Known pathogenic mutations in *APP*, *PSEN1,* or *PSEN2* were identified in 4 EOAD patients (0.4% of the entire cohort). Diagnosis and clinical symptoms were determined based on the diagnostic criteria of the National Institute of Neurological and Communicative Disorders and Stroke (NINCDS), the AD and Related Disorders Association (ADRDA) or the National Institute on Aging-Alzheimer’s Association (NIA-AA, (Hyman et al., 2012; McKhann et al., 1984; McKhann et al., 2011)). In addition, a neuropathological diagnosis of definite AD was available for 18 EOAD and 69 LOAD patients.

#### 2.1.3 Controls

To compare allelic frequencies and test for genetic associations, we also sequenced geographically matched, neurologically healthy individuals (n = 755, age at inclusion (AAI): 69.3 ± 8.9, range: 39–98, 67% female). At inclusion, controls were subjected to a Mini-Mental State Examination (MMSE) (score >24) or a Montreal Cognitive Assessment test (score > 26) (Folstein et al., 1975; Nasreddine et al., 2005). We also consulted the Healthy Exome (HEX) Database (https://www.alzforum.org/exomes/hex), which contains WES data for 478 neurologically healthy individuals above 60 years of age. One of the Exome Capture sequencing kits used in the HEX database was the same as the one used in-house, ensuring coverage of the regions of interest. This database was not included in any genetic association analysis. Characteristics for all cohorts are displayed in Table 1.

**TABLE 1:** Characteristics of the cohorts described in this study.

Status, n	Sex, female (%)	AAO/AAI ±SEM (range)	N with known mutations (%)
AD, 947			
EOAD, 262 LOAD, 685	148 (56.4) 457 (66.7)	59.1 ± 5.4 (37–65) 77.9 ± 5.8 (66–99)	4 (1.5) —
FTD, 335	161 (47.9)	62.9 ± 10.3 (29–85)	52 (15.6)
Controls, 755	506 (67)	69.3 ± 8.9 (39–98)	—
Subset, 426	309 (72.5)	67.9 ± 8.4 (43–96)	—

#### 2.1.4 Ethical approval

Research participants were included in the study after obtaining written informed consent. Ethics committees of all collaborating neurological centers approved the clinical study protocols and informed consent forms. The Ethics Committee of the University Hospital of Antwerp (UZA) and the University of Antwerp (Antwerp, Belgium) approved the genetic study protocols and informed consent forms.

### 2.2 Genetic screenings

We used WES data available for 209 FTD patients. WES was performed at the Neuromics Support Facility (NSF) of the VIB-UAntwerp Center for Molecular Neurology. DNA was sheared to the average size of 150 bp (Covaris) and libraries were prepared using the KAPA HyperPrep Kit (Roche). Four libraries were pooled equimolarly and exomes were captured using the SeqCap EZ Human Exome Kit v3.0 (Roche). Exomes were sequenced on the NextSeq500 platform using the NextSeq500 High output V2 kit (Illumina). We focused on a list of 289 brain-expressed miRNAs, based on previous research investigating miRNA variants associated with schizophrenia (Cammaerts et al., 2015b). After literature mining, this list was complemented with 4 additional miRNAs with possible involvement in FTD (miR-659, miR-132/212 cluster and miR-663) (Chen-Plotkin et al., 2012; Grasso et al., 2019; Rademakers et al., 2008). The probes of the SeqCap EZ Human Exome Kit v3.0 provided coverage for 263 miRNAs (Supplementary Table S1). Over all samples, on average 97.4% of the target region was sequenced at least at 20x coverage.

For FTD patients for whom no WES data was available (n = 126), we used an amplicon target amplification assay (Goossens et al., 2009) for the miRNAs harboring prioritized variants identified by WES. Briefly, multiplex polymerase chain reactions (PCR) were performed and purified using Agencourt AMPureXP beads (Beckman Coulter). Individual barcodes (Illumina Nextera XT) were introduced in a universal PCR step and samples were pooled, followed by massive parallel sequencing on a MiSeq platform (Illumina) at the NSF of the center.

Validation of the identified variants and sequencing of the AD cohort for *MIR885* variants was performed by Sanger sequencing on a 3730 DNA Analyzer (Applied Biosystems) using the BigDye Terminator Cycle Sequencing kit v3.1 (Applied Biosystems), followed by sequence analysis using SeqMan software (DNASTAR).

### 2.3 Bioinformatic analyses

For the analysis of whole exome and targeted datasets, we utilized a well-established in-house pipeline embedded in the GenomeComb package (v0.99) (Reumers et al., 2011). Briefly, after adapter clipping, reads were aligned to the reference genome hg19 assembly using Burrows-Wheeler Aligner MEMv0.7.15a (Li and Durbin, 2009). Realignment around indels was performed using GATKv3.8 UnifiedGenotyper. Following the removal of amplicon primers, variants were called and annotated using GATK and samtools (totalcoverage ≥ 5) (Li et al., 2009; McKenna et al., 2010). The resulting variant sets for every individual were combined, annotated and filtered (cut-offs: “coverage depth” > 20, “genotype quality” > 60 and “allelic ratio” > 1:3 (heterozygous) or 1:9 (homozygous) using GenomeComb (Reumers et al., 2011). Ultimately, we ended up with 192 unique miRNA variants in the FTD cohort.

**FIGURE 1:**
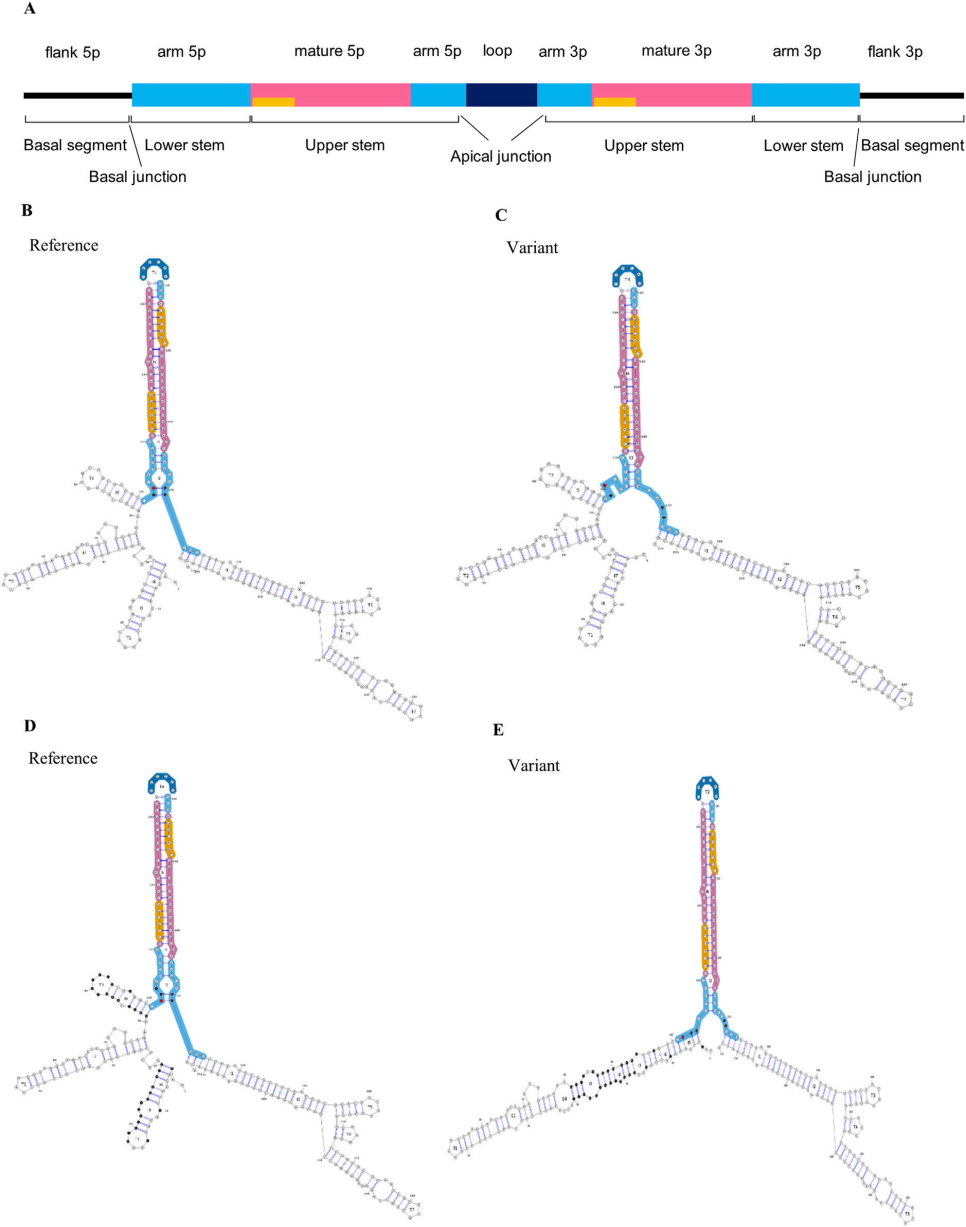
Impact of *MIR885* genetic variants on miRNA structure. **(A)** Linear representation and annotation of pri-miRNA. Above the bar are the annotations of miRNA regions based on the miRVaS tool. Yellow bars represent the seed sequence. Modified from Cammaerts et al., 2016. Below the bar are basic structural divisions based on DRCG8-Drosha processing: 1) The terminal loop, which is connected with main body of the miRNA via an apical junction, 2) upper stem which encompasses the mature miRNA sequence, 3) the lower stem which is separated from the upper stem after Drosha cleavage and 4) the basal segments, which are the single-stranded flanking regions following a basal junction at the end of the lower stem (Li et al., 2020; Ma et al., 2013). **(B–E)** Visual output of the predicted alterations in miR-885 secondary structure for the identified genetic variants. Minimum free energy (MFE) structures shown here represent the extent of changes for **(B, C)** rs897551430 and **(D, E)** rs993255773 variants. On the left are the secondary structures in the presence of the reference alleles **(B, D)**, and the changes in the presence of the mutant alleles are shown on the right **(C, E)**. Red dots indicate the variant locations. Nucleotides with altered base pairing are shown in black.

We prioritized WES variants based on their location within the miRNA and the minor allele frequency in public databases. Specifically, we confined our selection to rare variants (MAF <1%) in the Genome Aggregation (GnomAD) database v2.1.1 (Lek et al., 2016) residing in the miRNA seed region (Figure 1A), as it would have the most obvious impact on miRNA function. We proceeded to targeted resequencing of these miRNAs in additional FTD patients (n = 126) and a subset of 426 healthy controls (AAI: 67.9 ± 8.4, range: 43–96, 72.5% female) to determine frequencies of the identified variants in the FTD and control groups. Variants unique or with a higher MAF in controls compared to patients were considered benign and not investigated further. We used the miRVaS tool (http://mirvas.bioinf.be/, (Cammaerts et al., 2016)) to predict the impact of the identified variants on miRNA structure.

To test for genetic association of the rare *MIR885* variants (MAF <1%) with disease, we used the optimized sequence kernel association test (SKAT-O) which is suitable for small sample sizes (Lee et al., 2012). Due to the small genomic regions encoding miRNAs, rare variant association (statistical power = 0.8, significance level = 0.05) was performed only for the AD cohort (n = 947) against the entire control cohort (n = 755). The initial gene-burden analysis, as well as the covariate analysis with the integrations of sex and *APOE* genotype, were performed in R (version 4.3.2) using the SKAT package.

## 3 Results

### 3.1 Identification of miRNA variants in FTD patients

We identified 4 miRNA seed variants (MAF <5%) in 4 different miRNAs in WES data of FTD patients (n = 209, AAO: 65.2 ± 10.6) (Table 2), based on a list of brain-expressed miRNAs (Supplementary Table S1). The variants were present in 7 FTD patients. We then performed targeted resequencing of the 4 miRNAs in 126 additional FTD patients and 426 healthy controls. Results are shown in Table 3. In summary, we found the novel seed variant of *MIR656* in one more FTD patient. We did not find any of the seed variants of *MIR885, MIR656* and *MIR423* in control subjects. The seed variant of *MIR122* was identified in 7 additional patients (11 in total) and 8 controls. Subsequent case-control association analysis (chi-squared test) showed no significant differences in the calculated allelic frequencies (nominal significance = p > 0.05). In *MIR885*, we identified another variant (rs897551430) located in the arm region (Figure 1A). This variant was found in 2 FTD patients and was absent from controls. Other pathogenic mutations in the known causal FTD genes were excluded in the FTD patients carrying miRNA seed variants or the *MIR885* arm variant, except for one FTD patient carrying the *MIR122* variant together with a *C9orf72* repeat expansion. Different bioinformatic tools (TargetScan v.8 (http://www.targetscan.org/vert_80/, (Agarwal et al., 2015; McGeary et al., 2019)), miRDB (http://www.mirdb.org/, (Chen and Wang, 2020)) and miRmap v1.1 (https://mirmap.ezlab.org/, (Vejnar & Zdobnov, 2012)), predict that most miRNAs containing the identified variants bind the 3′ UTRs of causal or risk genes for frontotemporal lobar Degeneration. Next, we used miRNASNP v3 (https://guolab.wchscu.cn/miRNASNP/, (Liu et al., 2021)) to examine whether the presence of the seed variants would create novel miRNA binding sites or abrogate existing sites in 3′ UTRs. For the newly identified seed variant in *MIR656,* we used the miR2GO software (https://compbio.uthsc.edu/miR2GO/home.php), (Bhattacharya and Cui, 2015). Interestingly, the seed variants residing in *MIR656, MIR885* and *MIR423* are predicted to create potential binding sites for these miRNAs with the mRNA transcripts 2 and 3 of *C9orf72*, a major causal gene of FTLD (DeJesus-Hernandez et al., 2011; Gijselinck et al., 2012; Renton et al., 2011). The seed variant in *MIR423* also creates a binding site at the 3′ UTR of *FUS,* a gene genetically and functionally associated with FTD/ALS (Nolan et al., 2016; van Tartwijk et al., 2024).

**TABLE 2:** Rare miRNA seed variants in FTD patients.

Genomic position^a^	dbSNP153	miRNA	FTD carriers (MAF, n = 209, %)	GnomAD (MAF, European_non Finnish, %)
chr3: 10436198	rs941703617	*MIR885*	1 (0.24)	0.009
chr14: 101533070	—	*MIR656*	1 (0.24)	—
chr17: 28444118	rs766187585	*MIR423*	1 (0.24)	—
chr18: 56118358	rs41292412	*MIR122*	4 (0.95)	0.95

^a^ According to human reference sequence - Human Build 37/human genome 19.

**TABLE 3:** miRNA variants in FTD patients and controls.

Genomic position^a^	dbSNP153	Gene	miRNA location^b^	MAF in FTD patients (n = 335, %)	MAF in controls (n = 426, %)	GnomAD (MAF, European_non Finnish, %)
Patients (n = 335)						
chr3: 10436198	rs941703617	*MIR885*	Seed	0.15	—	0.0096
chr3: 10436244	rs897551430	*MIR885*	Arm	0.29	—	0.001
**chr14: 101533070**	—	***MIR656***	**Seed**	**0.29**	—	—
**chr17: 28444118**	**rs766187585**	***MIR423***	**Seed**	**0.15**	—	**0.002**
Controls (n = 426)						
chr3: 10436101	rs765699042	*MIR885*	Flank	—	0.11	0.04
chr17: 28444162	—	*MIR423*	Mature	—	0.11	—
chr18: 56118343	—	*MIR122*	Arm	—	0.11	—
Both groups						
**chr18: 56118358**	**rs41292412**	***MIR122***	**Seed**	**1.6**	**0.93**	**0.95**

^a^ According to human reference sequence - Human Build 37/human genome 19.

^b^ Region based on miRVaS annotation. Seed variants identified via WES are highlighted in bold.

### 3.2 Identification of MIR885 variants in the AD cohort

Interestingly, the miR-885-5p isoform was shown to be downregulated in the brain and serum of AD patients (Takousis et al., 2019; Tan et al., 2014). Therefore, we sequenced *MIR885* in the Flanders-Belgian AD cohort (n = 947) to investigate whether we could observe an enrichment of *MIR885* variants in AD patients.

We identified the arm variant rs897551430 in 4 LOAD patients and none of the control subjects. In addition, we found 2 rare variants, one in the arm region of the 5p isoform and one in the arm region of the 3p isoform. Each variant was present in 1 LOAD patient and was absent from controls (Table 4). All mutations were absent from the HEX database (https://www.alzforum.org/exomes/hex). Rare variant association analysis showed significant enrichment of *MIR885* variants in the AD cohort (SKAT-O p-value = 0.026). However, the association was lost after correcting for sex and *APOE* status (p-value = 0.16).

**TABLE 4:** Rare *MIR885* variants in AD patients.

Individual^a^	*APOE* status	AAO	Genomic position^b^	dbSNP153	miRNA location^c^	miRVaS prediction	MAF in AD patients (n = 947, %)	MAF in Controls (n = 755, %)	GnomAD (European_non Finnish, %)
AD1	34	87	chr3: 10436244	rs897551430	arm(5p)	Structural changes (5p-3p)	0.2	—	0.004%
AD2	34	76							
AD3	34	82							
AD4	24	74							
AD5	34	86	chr3: 10436245	rs993255773	arm(5p)	Structural changes (5p-3p)	0.05	—	0.002%
AD6	44	70	chr3: 10436207	—	arm(3p)	No changes	0.05	—	—

^a^ No pathogenic mutations were found in any of the *MIR885* variant carriers.

^b^ According to human reference sequence - Human Build 37/human genome 19.

^c^ Region based on miRVaS annotation.

### 3.3 Structural changes induced by miRNA variants

We assessed the impact of the *MIR885* variants on the secondary structure using miRVaS. While no changes were observed for the variant in the 3p isoform, variants rs897551430 and rs993255773 evoke significant changes in the hairpin structure of the pre-miRNA (Table 4; Figures 1C, E), which are also predicted to strongly reduce mature miR-885 levels. Interestingly, structural changes occur to both isoforms, which could be attributed to the altered base pairing in the presence of the mutant alleles (Figures 1C, E; black dots). Structural changes were also observed in presence of the *MIR656* seed variant, identified in 2 FTD patients (Supplementary Figure S1).

## 4 Discussion

By now, miRNAs are well known to play a critical role in brain development and functions of diverse neuronal populations (Budde et al., 2010; Tan et al., 2013). miRNA dysregulation in the brain results in impaired molecular pathways that are shared by NBDs, such as AD and PD (reviewed in (Sadlon et al., 2019)). Genetic variants can lead to impairment of miRNA functions via different mechanisms, but there is still limited evidence associating miRNA variants with brain neurodegeneration.

**FIGURE 2:**
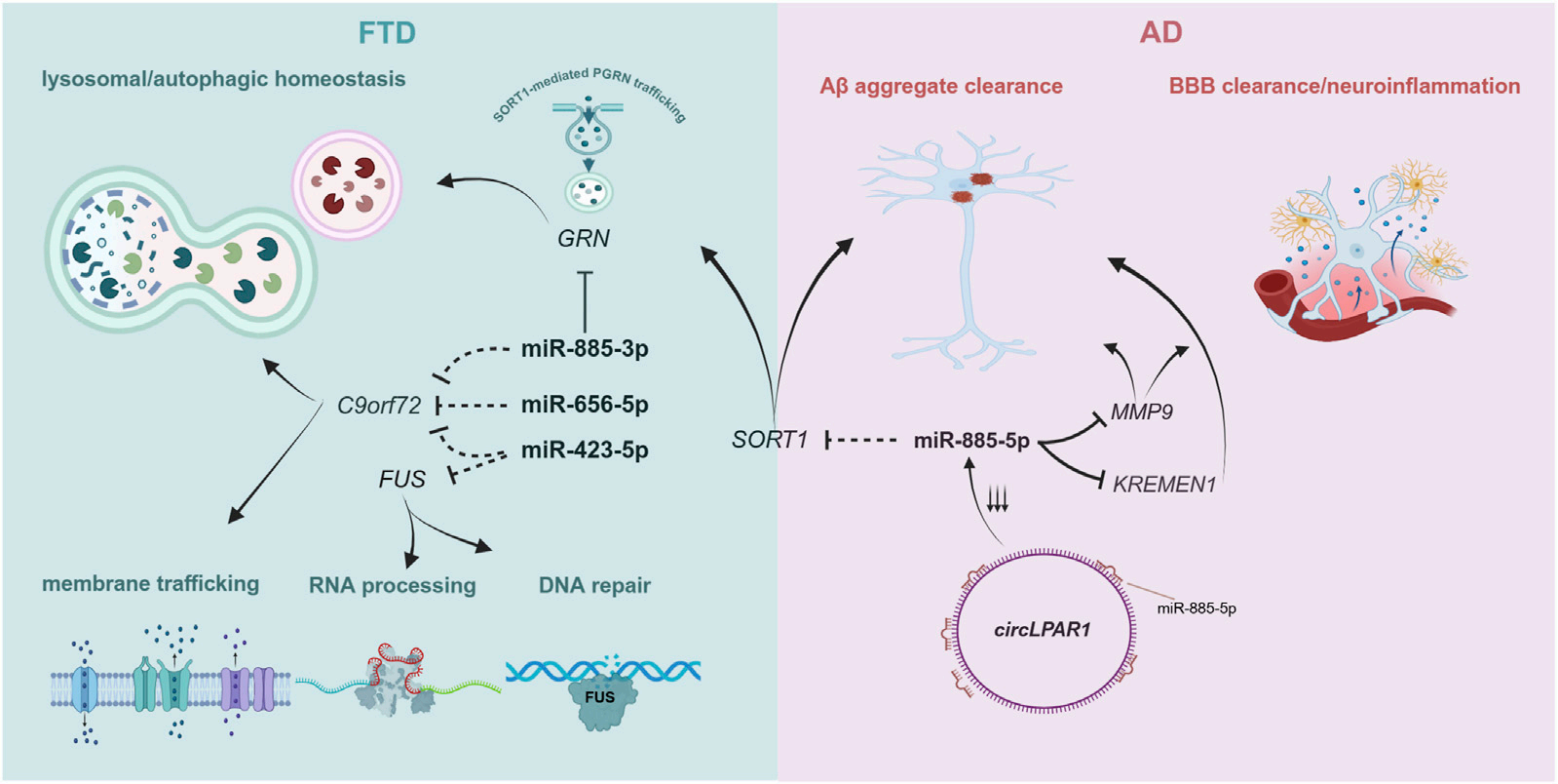
Molecular processes involving FTD- and AD-associated genes targeted by the miRNAs described in this study. Dotted inhibition lines correspond to predicted targets for each miRNA, while full black inhibition lines represent experimentally validated targets. Given the predominant function of miRNAs in repressing gene expression, we included molecular pathways that are affected by depleted levels of these genes. Created by biorender.com.

For this study, we investigated the possible involvement of miRNA variants in the pathogenicity of FTD and AD. We identified 4 rare seed variants in 4 miRNAs (*MIR122, MIR656, MIR423, MIR885*) in our FTD cohort. Although rare variant association analysis was not feasible due to the small sample size, the presence of such rare variants in our patient cohort could indicate an involvement of these miRNAs in neurodegenerative processes. Indeed, bioinformatic analyses suggest functional implications of these miRNAs/variants related to FTD. miR-885-3p is predicted to bind the 3′ UTR of *GRN* by both miRmap and TargetScan and this is also validated by HITS-CLIP performed in the human brain cortex (Boudreau et al., 2014). This interaction warrants functional investigation in a FTD context to elucidate whether progranulin (PGRN) protein levels could be downregulated by miR-885-3p, which would align with the described haploinsufficiency mechanism linking *GRN* loss-of-function mutations with FTD. We also reported seed variants creating new miRNA binding sites at the 3′ UTRs of *C9orf72 (MIR423, MIR656, MIR856)* and *FUS (MIR423),* with a significant probability score (miRmap, P.over exact <0.05). FUS plays a critical role in RNA processing and DNA repair, while C9ORF72 haploinsufficiency has been linked with ALS/FTD via impaired membrane trafficking and autophagic function (Pang and Hu, 2021; Shi et al., 2018). These observations need to be interpreted cautiously as predictions need to be experimentally validated. Further, expression patterns for each miRNA-mRNA interaction must be investigated in disease-related tissue to confirm biological relevance. Nonetheless, our genetic and bioinformatic approach suggests miRNA-mediated molecular processes that could contribute to FTD pathogenicity (Figure 2).

*MIR885* is the most intriguing case. Both the 3p and the 5p isoform are enriched in brain (Human miRNA tissue atlas (https://ccb-web.cs.uni-saarland.de/tissueatlas2, (Keller et al., 2021; Ludwig et al., 2016)), suggesting a distinct function for *MIR885* in brain. The 5p isoform is the leading strand, which is quite common for mature miRNAs derived from the same precursor (McCall et al., 2017). miR-885-5p is predicted to bind the *SORT1* gene, encoding sortilin 1, with very strong affinity metrics (TargetScan v.8, miRDB and miRmap v.1.1). Sortilin 1 is a known regulator of PGRN levels, as it mediates PGRN lysosomal trafficking, leading to reduced extracellular PGRN levels (Hu et al., 2010). Common variants adjacent to the *SORT1* gene have been associated with reduced PGRN levels in plasma in control subjects and FTD patients (Carrasquillo et al., 2010), while rare *SORT1* variants have been associated with increased risk for FTD in different populations (Philtjens et al., 2018). Also, a recent GWAS identified a *SORT1* missense mutation as the sentinel SNP in a novel risk locus for AD (Bellenguez et al., 2022). Involvement of miR-885-5p in disease pathogenesis could be investigated via direct interaction with *SORT1*, where altered sortilin 1 expression might dysregulate unique or shared pathways between FTD and AD leading to neurodegeneration. Another study described the miR-885-5p interaction with matrix metalloprotease 9 (MMP9) in glioma cells (Yan et al., 2011). MMP9 is a potential AD biomarker for disease progression, with higher serum levels of MMP9 correlated with faster cognitive decline in patients with mild cognitive impairment attributed to AD (Abe et al., 2020). This is aligned with another study, which identified higher MMP9 levels in the brain of AD patients with a later Braak stage (Hernandes-Alejandro et al., 2020). Taken together, a potential involvement of miR-885-5p in AD could be attributed to its interaction with MMP9.

Both SORT1 and MMP9 are reported to assist in the degradation and clearance of toxic Αβ aggregates (Radosinska and Radosinska, 2025; Ruan et al., 2018). However, their neuroprotective function can be attenuated by the presence of APOE Ε4 (Asaro et al., 2020; Shackleton et al., 2019). Therefore, any mechanistic impact derived from their interaction with miR-885-5p could be masked by the inarguably higher effect in the presence of an APOE E4 isoform. A recent study showcased that circ_0003611 (*circLPAR1*), a circular RNA upregulated in AD (Li et al., 2020), acts as a sponge for miR-885-5p (Pan et al., 2022). Downregulation of *circPARL1* significantly reduced Αβ-induced apoptosis, inflammation and oxidative stress in a human neuroblastoma cell line (SK-N-SH cells) (Pan et al., 2022). A similar effect was observed in Aβ-treated SK-N-SH cells after exogenous miR-885-5p administration. This mitigation of Αβ-induced pathology was attributed to miR-885-5p binding and reducing transcript levels of KREMEN1, a gene previously implicated in AD via noncoding RNA interactions (Wang et al., 2019). These results further support a protective role for miR-885-5p in AD, independent of APOE status, via alleviating Aβ-induced inflammation, which appears to be exacerbated when miR-885-5p levels are depleted (Figure 2). Investigation of miR-885-5p function in a knockdown model using neuronal-like cell types would provide valuable insights of its implication in neurodegenerative processes.

We identified 2 ultra-rare *MIR885* variants (rs941703617, rs897551430) in 3 FTD patients (Table 3), both absent from control subjects and the HEX database (https://www.alzforum.org/exomes/hex). Extending our screening in our AD cohort, we identified rs897551430 in 4 LOAD patients, as well as two other rare variants in 2 LOAD patients (Table 4). Variants rs897551430 and rs993255773 are predicted to strongly decrease pre-miRNA-885 levels (miRNASNP v3, http://bioinfo.life.hust.edu.cn/miRNASNP/). This is also supported by the prominent changes caused to the secondary miRNA structure in the presence of each mutant allele (Figure 1). Both variants are located at the 5′ end of the 5p isoform, which corresponds to the lower stem and could thus affect processing of the pri-miRNA by the DGCR8-Drosha complex (Bofill-De Ros et al., 2019; Ma et al., 2013). Rare variant association analysis initially showed a significant enrichment for *MIR885* variants in our AD cohort (SKAT-O p-value = 0.026). However, upon the integration of sex and *APOE* genotypes as covariates, the genetic association was lost. Genetic studies on rare variants are inherently challenging. A recent study performed region-based rare variant association analysis using whole-genome sequencing data of >6,000 amyotrophic lateral sclerosis (ALS) patients and >70,000 healthy controls to explore the noncoding genome (Eitan et al., 2022). Despite the large cohort size, no disease association was found for any of the identified miRNA variants. This was, partially, attributed to the small genomic size of miRNAs (∼120 nucleotides), which significantly hinders gene-burden analysis for rare variants, as it requires a dramatic increase in sample size to prevent missing risk or potentially causal genetic variation. The importance of using large population datasets was also underscored in the most recent phenome-wide association study which used genetic and clinical data from over 400,000 participants of the UK BioBank and showcased the pleiotropic effect of common miRNA variants (Mustafa et al., 2023). That said, we believe that the observed genetic enrichment of ultra-rare variants before *APOE* status and sex correction in our relatively small cohort size endorses genetic screening in extended cohorts or integration of publicly available AD and FTD genetic data to substantially increase the power to test for a possible association with disease risk.

In conclusion, we identified rare genetic variants of brain-expressed miRNAs in patients with NBDs. Functional investigation for all variants is warranted, as they are predicted to target disease-associated genes. Elucidation of miRNA function in neurodegeneration will pave the way for novel therapeutic approaches for brain disorders. Furthermore, given that miRNAs constitute robust biomarkers, establishing miRNA expression profiles in a disease-related context could offer opportunities for earlier and improved differential diagnosis. We believe that the genetic findings presented for *MIR885* suggest an implication for this miRNA gene in AD pathology and underscore the disease relevance of genetic variation in noncoding genomic regions.

## Data Availability

The raw data supporting the conclusions of this article will be made available by the authors, without undue reservation.

## References

[B1] Abe K. Chiba Y. Hattori S. Yoshimi A. Asami T. Katsuse O. (2020). Influence of plasma matrix metalloproteinase levels on longitudinal changes in Alzheimer's disease (AD) biomarkers and cognitive function in patients with mild cognitive impairment due to AD registered in the Alzheimer's Disease Neuroimaging Initiative database. J. Neurol. Sci. 416, 116989. 10.1016/j.jns.2020.116989 32603972

[B2] Agarwal V. Bell G. W. Nam J. W. Bartel D. P. (2015). Predicting effective microRNA target sites in mammalian mRNAs. Elife 4, e05005. 10.7554/eLife.05005 26267216 PMC4532895

[B3] Asaro A. Carlo-Spiewok A. S. Malik A. R. Rothe M. Schipke C. G. Peters O. (2020). Apolipoprotein E4 disrupts the neuroprotective action of sortilin in neuronal lipid metabolism and endocannabinoid signaling. Alzheimers Dement. 16 (9), 1248–1258. 10.1002/alz.12121 32588544

[B4] Bellenguez C. Kucukali F. Jansen I. E. Kleineidam L. Moreno-Grau S. Amin N. (2022). New insights into the genetic etiology of Alzheimer's disease and related dementias. Nat. Genet. 54 (4), 412–436. 10.1038/s41588-022-01024-z 35379992 PMC9005347

[B5] Bhattacharya A. Cui Y. (2015). miR2GO: comparative functional analysis for microRNAs. Bioinformatics 31 (14), 2403–2405. 10.1093/bioinformatics/btv140 25762653

[B6] Bofill-De Ros X. Kasprzak W. K. Bhandari Y. Fan L. Cavanaugh Q. Jiang M. (2019). Structural differences between pri-miRNA paralogs promote alternative Drosha cleavage and expand target repertoires. Cell Rep. 26 (2), 447–459 e444. 10.1016/j.celrep.2018.12.054 30625327 PMC6369706

[B7] Boudreau R. L. Jiang P. Gilmore B. L. Spengler R. M. Tirabassi R. Nelson J. A. (2014). Transcriptome-wide discovery of microRNA binding sites in human brain. Neuron 81 (2), 294–305. 10.1016/j.neuron.2013.10.062 24389009 PMC4108341

[B8] Budde H. Schmitt S. Fitzner D. Opitz L. Salinas-Riester G. Simons M. (2010). Control of oligodendroglial cell number by the miR-17-92 cluster. Development 137 (13), 2127–2132. 10.1242/dev.050633 20504959

[B9] Cammaerts S. Strazisar M. De Rijk P. Del Favero J. (2015a). Genetic variants in microRNA genes: impact on microRNA expression, function, and disease. Front. Genet. 6, 186. 10.3389/fgene.2015.00186 26052338 PMC4439572

[B10] Cammaerts S. Strazisar M. Dierckx J. Del Favero J. De Rijk P. (2016). miRVaS: a tool to predict the impact of genetic variants on miRNAs. Nucleic Acids Res. 44 (3), e23. 10.1093/nar/gkv921 26384425 PMC4756848

[B11] Cammaerts S. Strazisar M. Smets B. Weckhuysen S. Nordin A. De Jonghe P. (2015b). Schizophrenia-associated MIR204 regulates noncoding RNAs and affects neurotransmitter and ion channel gene sets. PLoS One 10 (12), e0144428. 10.1371/journal.pone.0144428 26714269 PMC4695081

[B12] Carrasquillo M. M. Nicholson A. M. Finch N. Gibbs J. R. Baker M. Rutherford N. J. (2010). Genome-wide screen identifies rs646776 near sortilin as a regulator of progranulin levels in human plasma. Am. J. Hum. Genet. 87 (6), 890–897. 10.1016/j.ajhg.2010.11.002 21087763 PMC2997361

[B13] Chen Y. Wang X. (2020). miRDB: an online database for prediction of functional microRNA targets. Nucleic Acids Res. 48 (D1), D127-D131–D131. 10.1093/nar/gkz757 31504780 PMC6943051

[B14] Chen-Plotkin A. S. Unger T. L. Gallagher M. D. Bill E. Kwong L. K. Volpicelli-Daley L. (2012). TMEM106B, the risk gene for frontotemporal dementia, is regulated by the microRNA-132/212 cluster and affects progranulin pathways. J. Neurosci. 32 (33), 11213–11227. 10.1523/JNEUROSCI.0521-12.2012 22895706 PMC3446826

[B15] DeJesus-Hernandez M. Mackenzie I. R. Boeve B. F. Boxer A. L. Baker M. Rutherford N. J. (2011). Expanded GGGGCC hexanucleotide repeat in noncoding region of C9ORF72 causes chromosome 9p-linked FTD and ALS. Neuron 72 (2), 245–256. 10.1016/j.neuron.2011.09.011 21944778 PMC3202986

[B16] Eitan C. Siany A. Barkan E. Olender T. van Eijk K. R. Moisse M. (2022). Whole-genome sequencing reveals that variants in the interleukin 18 receptor accessory protein 3'UTR protect against ALS. Nat. Neurosci. 25 (4), 433–445. 10.1038/s41593-022-01040-6 35361972 PMC7614916

[B17] Folstein M. F. Folstein S. E. McHugh P. R. (1975). Mini-mental state. A practical method for grading the cognitive state of patients for the clinician. J. Psychiatr. Res. 12 (3), 189–198. 10.1016/0022-3956(75)90026-6 1202204

[B18] Freischmidt A. Muller K. Zondler L. Weydt P. Mayer B. von Arnim C. A. (2015). Serum microRNAs in sporadic amyotrophic lateral sclerosis. Neurobiol. Aging 36 (9), 2660 e2615–e20. 10.1016/j.neurobiolaging.2015.06.003 26142125

[B19] Ghanbari M. Darweesh S. K. de Looper H. W. van Luijn M. M. Hofman A. Ikram M. A. (2016a). Genetic variants in MicroRNAs and their binding sites are associated with the risk of Parkinson disease. Hum. Mutat. 37 (3), 292–300. 10.1002/humu.22943 26670097

[B20] Ghanbari M. Ikram M. A. de Looper H. W. J. Hofman A. Erkeland S. J. Franco O. H. (2016b). Genome-wide identification of microRNA-related variants associated with risk of Alzheimer's disease. Sci. Rep. 6, 28387. 10.1038/srep28387 27328823 PMC4916596

[B21] Gijselinck I. Van Langenhove T. van der Zee J. Sleegers K. Philtjens S. Kleinberger G. (2012). A C9orf72 promoter repeat expansion in a Flanders-Belgian cohort with disorders of the frontotemporal lobar degeneration-amyotrophic lateral sclerosis spectrum: a gene identification study. Lancet Neurol. 11 (1), 54–65. 10.1016/S1474-4422(11)70261-7 22154785

[B22] Goossens D. Moens L. N. Nelis E. Lenaerts A. S. Glassee W. Kalbe A. (2009). Simultaneous mutation and copy number variation (CNV) detection by multiplex PCR-based GS-FLX sequencing. Hum. Mutat. 30 (3), 472–476. 10.1002/humu.20873 19058222

[B69] Gorno-Tempini M. L. Hillis A. E. Weintraub S. Kertesz A. Mendez M. Cappa S. F. (2011). Classification of primary progressive aphasia and its variants. Neurology 76 (11), 1006–1014. 10.1212/WNL.0b013e31821103e6 21325651 PMC3059138

[B23] Grasso M. Piscopo P. Talarico G. Ricci L. Crestini A. Tosto G. (2019). Plasma microRNA profiling distinguishes patients with frontotemporal dementia from healthy subjects. Neurobiol. Aging 84, e241–e240. 10.1016/j.neurobiolaging.2019.01.024 30826067

[B24] Hernandes-Alejandro M. Montano S. Harrington C. R. Wischik C. M. Salas-Casas A. Cortes-Reynosa P. (2020). Analysis of the relationship between metalloprotease-9 and tau protein in Alzheimer’s disease. J. Alzheimers Dis. 76 (2), 553–569. 10.3233/JAD-200146 32538846

[B25] Hu F. Padukkavidana T. Vaegter C. B. Brady O. A. Zheng Y. Mackenzie I. R. (2010). Sortilin-mediated endocytosis determines levels of the frontotemporal dementia protein, progranulin. Neuron 68 (4), 654–667. 10.1016/j.neuron.2010.09.034 21092856 PMC2990962

[B26] Hyman B. T. Phelps C. H. Beach T. G. Bigio E. H. Cairns N. J. Carrillo M. C. (2012). National Institute on Aging-Alzheimer’s Association guidelines for the neuropathologic assessment of Alzheimer's disease. Alzheimers Dement. 8 (1), 1–13. 10.1016/j.jalz.2011.10.007 22265587 PMC3266529

[B27] Junn E. Lee K. W. Jeong B. S. Chan T. W. Im J. Y. Mouradian M. M. (2009). Repression of alpha-synuclein expression and toxicity by microRNA-7. Proc. Natl. Acad. Sci. U. S. A. 106 (31), 13052–13057. 10.1073/pnas.0906277106 19628698 PMC2722353

[B28] Keller A. Groger L. Tschernig T. Solomon J. Laham O. Schaum N. (2021). miRNATissueAtlas2: an update to the human miRNA tissue atlas. Nucleic Acids Res. 50, D211–D221. 10.1093/nar/gkab808 PMC8728130 34570238

[B29] Landgraf P. Rusu M. Sheridan R. Sewer A. Iovino N. Aravin A. (2007). A mammalian microRNA expression atlas based on small RNA library sequencing. Cell 129 (7), 1401–1414. 10.1016/j.cell.2007.04.040 17604727 PMC2681231

[B30] Lee S. Emond M. J. Bamshad M. J. Barnes K. C. Rieder M. J. Nickerson D. A. (2012). Optimal unified approach for rare-variant association testing with application to small-sample case-control whole-exome sequencing studies. Am. J. Hum. Genet. 91 (2), 224–237. 10.1016/j.ajhg.2012.06.007 22863193 PMC3415556

[B31] Lek M. Karczewski K. J. Minikel E. V. Samocha K. E. Banks E. Fennell T. (2016). Analysis of protein-coding genetic variation in 60,706 humans. Nature 536 (7616), 285–291. 10.1038/nature19057 27535533 PMC5018207

[B32] Li H. Durbin R. (2009). Fast and accurate short read alignment with Burrows-Wheeler transform. Bioinformatics 25 (14), 1754–1760. 10.1093/bioinformatics/btp324 19451168 PMC2705234

[B33] Li H. Handsaker B. Wysoker A. Fennell T. Ruan J. Homer N. (2009). The sequence alignment/map format and SAMtools. Bioinformatics 25 (16), 2078–2079. 10.1093/bioinformatics/btp352 19505943 PMC2723002

[B34] Li S. Nguyen T. D. Nguyen T. L. Nguyen T. A. (2020). Mismatched and wobble base pairs govern primary microRNA processing by human Microprocessor. Nat. Commun. 11 (1), 1926. 10.1038/s41467-020-15674-2 32317642 PMC7174388

[B35] Li Y. Fan H. Sun J. Ni M. Zhang L. Chen C. (2020). Circular RNA expression profile of Alzheimer’s disease and its clinical significance as biomarkers for the disease risk and progression. Int. J. Biochem. Cell Biol. 123, 105747. 10.1016/j.biocel.2020.105747 32315771

[B36] Liu C. J. Fu X. Xia M. Zhang Q. Gu Z. Guo A. Y. (2021). miRNASNP-v3: a comprehensive database for SNPs and disease-related variations in miRNAs and miRNA targets. Nucleic Acids Res. 49 (D1), D1276–D1281. 10.1093/nar/gkaa783 32990748 PMC7778889

[B37] Ludwig N. Leidinger P. Becker K. Backes C. Fehlmann T. Pallasch C. (2016). Distribution of miRNA expression across human tissues. Nucleic Acids Res. 44 (8), 3865–3877. 10.1093/nar/gkw116 26921406 PMC4856985

[B38] Ma H. Wu Y. Choi J. G. Wu H. (2013). Lower and upper stem-single-stranded RNA junctions together determine the Drosha cleavage site. Proc. Natl. Acad. Sci. U. S. A. 110 (51), 20687–20692. 10.1073/pnas.1311639110 24297910 PMC3870748

[B39] McCall M. N. Kim M. S. Adil M. Patil A. H. Lu Y. Mitchell C. J. (2017). Toward the human cellular microRNAome. Genome Res. 27 (10), 1769–1781. 10.1101/gr.222067.117 28877962 PMC5630040

[B40] McGeary S. E. Lin K. S. Shi C. Y. Pham T. M. Bisaria N. Kelley G. M. (2019). The biochemical basis of microRNA targeting efficacy. Science 366 (6472), eaav1741. 10.1126/science.aav1741 31806698 PMC7051167

[B41] McKenna A. Hanna M. Banks E. Sivachenko A. Cibulskis K. Kernytsky A. (2010). The Genome Analysis Toolkit: a MapReduce framework for analyzing next-generation DNA sequencing data. Genome Res. 20 (9), 1297–1303. 10.1101/gr.107524.110 20644199 PMC2928508

[B42] McKhann G. Drachman D. Folstein M. Katzman R. Price D. Stadlan E. M. (1984). Clinical diagnosis of Alzheimer’s disease: report of the NINCDS-ADRDA work group under the auspices of department of health and human services task force on Alzheimer's disease. Neurology 34 (7), 939–944. 10.1212/wnl.34.7.939 6610841

[B43] McKhann G. M. Knopman D. S. Chertkow H. Hyman B. T. Jack C. R. Jr. Kawas C. H. (2011). The diagnosis of dementia due to Alzheimer’s disease: recommendations from the National Institute on Aging-Alzheimer’s Association workgroups on diagnostic guidelines for Alzheimer's disease. Alzheimers Dement. 7 (3), 263–269. 10.1016/j.jalz.2011.03.005 21514250 PMC3312024

[B44] Mustafa R. Ghanbari M. Karhunen V. Evangelou M. Dehghan A. (2023). Phenome-wide association study on miRNA-related sequence variants: the UK Biobank. Hum. Genomics 17 (1), 104. 10.1186/s40246-023-00553-w 37996941 PMC10668386

[B45] Nasreddine Z. S. Phillips N. A. Bedirian V. Charbonneau S. Whitehead V. Collin I. (2005). The Montreal Cognitive Assessment, MoCA: a brief screening tool for mild cognitive impairment. J. Am. Geriatr. Soc. 53 (4), 695–699. 10.1111/j.1532-5415.2005.53221.x 15817019

[B46] Nolan M. Talbot K. Ansorge O. (2016). Pathogenesis of FUS-associated ALS and FTD: insights from rodent models. Acta Neuropathol. Commun. 4 (1), 99. 10.1186/s40478-016-0358-8 27600654 PMC5011941

[B47] Pan W. Hu Y. Wang L. Li J. (2022). Circ_0003611 acts as a miR-885-5p sponge to aggravate the amyloid-β-induced neuronal injury in Alzheimer’s disease. Metab. Brain Dis. 37 (4), 961–971. 10.1007/s11011-022-00912-x 35076819

[B48] Pang W. Hu F. (2021). Cellular and physiological functions of C9ORF72 and implications for ALS/FTD. J. Neurochem. 157 (3), 334–350. 10.1111/jnc.15255 33259633 PMC8842544

[B49] Philtjens S. Van Mossevelde S. van der Zee J. Wauters E. Dillen L. Vandenbulcke M. (2018). Rare nonsynonymous variants in SORT1 are associated with increased risk for frontotemporal dementia. Neurobiol. Aging, 66(181), 181.e3-181.e10. 10.1016/j.neurobiolaging.2018.02.011 29555433

[B50] Rademakers R. Eriksen J. L. Baker M. Robinson T. Ahmed Z. Lincoln S. J. (2008). Common variation in the miR-659 binding-site of GRN is a major risk factor for TDP43-positive frontotemporal dementia. Hum. Mol. Genet. 17 (23), 3631–3642. 10.1093/hmg/ddn257 18723524 PMC2581433

[B51] Radosinska D. Radosinska J. (2025). The link between matrix metalloproteinases and Alzheimer's disease pathophysiology. Mol. Neurobiol. 62 (1), 885–899. 10.1007/s12035-024-04315-0 38935232 PMC11711632

[B70] Rascovsky K. Hodges J. R. Knopman D. Mendez M. F. Kramer J. H. Neuhaus J. (2011). Sensitivity of revised diagnostic criteria for the behavioural variant of frontotemporal dementia. Brain 134 (Pt 9), 2456–2477. 10.1093/brain/awr179 21810890 PMC3170532

[B52] Renton A. E. Majounie E. Waite A. Simon-Sanchez J. Rollinson S. Gibbs J. R. (2011). A hexanucleotide repeat expansion in C9ORF72 is the cause of chromosome 9p21-linked ALS-FTD. Neuron 72 (2), 257–268. 10.1016/j.neuron.2011.09.010 21944779 PMC3200438

[B53] Reumers J. De Rijk P. Zhao H. Liekens A. Smeets D. Cleary J. (2011). Optimized filtering reduces the error rate in detecting genomic variants by short-read sequencing. Nat. Biotechnol. 30 (1), 61–68. 10.1038/nbt.2053 22178994

[B54] Ruan C. S. Liu J. Yang M. Saadipour K. Zeng Y. Q. Liao H. (2018). Sortilin inhibits amyloid pathology by regulating non-specific degradation of APP. Exp. Neurol. 299 (Pt A), 75–85. 10.1016/j.expneurol.2017.10.018 29056359

[B55] Sadlon A. Takousis P. Alexopoulos P. Evangelou E. Prokopenko I. Perneczky R. (2019). miRNAs identify shared pathways in Alzheimer’s and Parkinson’s diseases. Trends Mol. Med. 25 (8), 662–672. 10.1016/j.molmed.2019.05.006 31221572

[B56] Schulz J. Takousis P. Wohlers I. Itua I. O. G. Dobricic V. Rucker G. (2019). Meta-analyses identify differentially expressed micrornas in Parkinson's disease. Ann. Neurol. 85 (6), 835–851. 10.1002/ana.25490 30990912

[B57] Shackleton B. Ringland C. Abdullah L. Mullan M. Crawford F. Bachmeier C. (2019). Influence of matrix metallopeptidase 9 on beta-amyloid elimination across the blood-brain barrier. Mol. Neurobiol. 56 (12), 8296–8305. 10.1007/s12035-019-01672-z 31209784 PMC6842100

[B58] Sheinerman K. S. Toledo J. B. Tsivinsky V. G. Irwin D. Grossman M. Weintraub D. (2017). Circulating brain-enriched microRNAs as novel biomarkers for detection and differentiation of neurodegenerative diseases. Alzheimers Res. Ther. 9 (1), 89. 10.1186/s13195-017-0316-0 29121998 PMC5679501

[B59] Shi Y. Lin S. Staats K. A. Li Y. Chang W. H. Hung S. T. (2018). Haploinsufficiency leads to neurodegeneration in C9ORF72 ALS/FTD human induced motor neurons. Nat. Med. 24 (3), 313–325. 10.1038/nm.4490 29400714 PMC6112156

[B60] Su L. Wang C. Zheng C. Wei H. Song X. (2018). A meta-analysis of public microarray data identifies biological regulatory networks in Parkinson’s disease. BMC Med. Genomics 11 (1), 40. 10.1186/s12920-018-0357-7 29653596 PMC5899355

[B61] Takousis P. Sadlon A. Schulz J. Wohlers I. Dobricic V. Middleton L. (2019). Differential expression of microRNAs in Alzheimer’s disease brain, blood, and cerebrospinal fluid. Alzheimers Dement. 15 (11), 1468–1477. 10.1016/j.jalz.2019.06.4952 31495604

[B62] Tan C. L. Plotkin J. L. Veno M. T. von Schimmelmann M. Feinberg P. Mann S. (2013). MicroRNA-128 governs neuronal excitability and motor behavior in mice. Science 342 (6163), 1254–1258. 10.1126/science.1244193 24311694 PMC3932786

[B63] Tan L. Yu J. T. Tan M. S. Liu Q. Y. Wang H. F. Zhang W. (2014). Genome-wide serum microRNA expression profiling identifies serum biomarkers for Alzheimer’s disease. J. Alzheimers Dis. 40 (4), 1017–1027. 10.3233/JAD-132144 24577456

[B64] van Tartwijk F. W. Wunderlich L. C. S. Mela I. Makarchuk S. Jakobs M. A. H. Qamar S. (2024). Mutation of the ALS-/FTD-Associated RNA-binding protein FUS affects axonal development. J. Neurosci. 44 (27), e2148232024. 10.1523/JNEUROSCI.2148-23.2024 38692734 PMC7616130

[B65] Vejnar C. E. Zdobnov E. M. (2012). MiRmap: comprehensive prediction of microRNA target repression strength. Nucleic Acids Res. 40 (22), 11673–11683. 10.1093/nar/gks901 23034802 PMC3526310

[B66] Wang H. Lu B. Chen J. (2019). Knockdown of lncRNA SNHG1 attenuated Aβ25-35-inudced neuronal injury via regulating KREMEN1 by acting as a ceRNA of miR-137 in neuronal cells. Biochem. Biophys. Res. Commun. 518 (3), 438–444. 10.1016/j.bbrc.2019.08.033 31447119

[B67] Yan W. Zhang W. Sun L. Liu Y. You G. Wang Y. (2011). Identification of MMP-9 specific microRNA expression profile as potential targets of anti-invasion therapy in glioblastoma multiforme. Brain Res. 1411, 108–115. 10.1016/j.brainres.2011.07.002 21831363

[B68] Zeng Y. Yi R. Cullen B. R. (2005). Recognition and cleavage of primary microRNA precursors by the nuclear processing enzyme Drosha. EMBO J. 24 (1), 138–148. 10.1038/sj.emboj.7600491 15565168 PMC544904

