# Supplementary material for: Investigation of the role of miRNA variants in neurodegenerative brain diseases
Source: Front Genet. 2025 Feb 26;16:1506169. doi: 10.3389/fgene.2025.1506169 (PMC11897046; doi:10.3389/fgene.2025.1506169)
Supplement: Supplementary file 1 [file Table1.docx]

**Supplementary materials**

***Supplementary Tables and Figures***

**S1 Table. Genomic position of the brain-expressed miRNAs targeted by whole exome sequencing.**

| **chromosome** | **begin^a^** | **end** | **miRNA^b^** |
| --- | --- | --- | --- |
| 1 | 1102452 | 1102760 | mir-200b |
| 1 | 1103209 | 1103368 | mir-200a |
| 1 | 1104341 | 1104558 | mir-429 |
| 1 | 3477187 | 3477429 | mir-551a |
| 1 | 41219982 | 41220142 | mir-30e |
| 1 | 41222920 | 41223220 | mir-30c-1 |
| 1 | 65524063 | 65524285 | mir-101-1 |
| 1 | 68649108 | 68649348 | mir-1262 |
| 1 | 71533103 | 71533423 | mir-186 |
| 1 | 94312363 | 94312593 | mir-760 |
| 1 | 98511545 | 98511888 | mir-137 |
| 1 | 110141412 | 110141689 | mir-197 |
| 1 | 117214162 | 117214488 | mir-320b-1 |
| 1 | 155164907 | 155165159 | mir-92b |
| 1 | 156390038 | 156390352 | mir-9-1 |
| 1 | 172107902 | 172108068 | mir-214 |
| 1 | 172113652 | 172113894 | mir-199a-2 |
| 1 | 176998390 | 176998736 | mir-488 |
| 1 | 193105465 | 193105758 | mir-1278 |
| 1 | 198827919 | 198828470 | mir-181b-1 |
| 1 | 205417331 | 205417627 | mir-135b |
| 1 | 207975097 | 207975329 | mir-29c |
| 1 | 207975599 | 207975905 | mir-29b-2 |
| 1 | 220291444 | 220291612 | mir-194-1 |
| 2 | 25551401 | 25551633 | mir-1301 |
| 2 | 56210076 | 56210403 | mir-217 |
| 2 | 56216046 | 56216325 | mir-216a |
| 2 | 56227704 | 56227975 | mir-216b |
| 2 | 136422929 | 136423182 | mir-128-1 |
| 2 | 180725391 | 180725702 | mir-1258 |
| 2 | 219267198 | 219267516 | mir-26b |
| 2 | 219866330 | 219866468 | mir-375 |
| 2 | 220158745 | 220159007 | mir-153-1 |
| 2 | 241395382 | 241395606 | mir-149 |
| 3 | 10436037 | 10436272 | mir-885 |
| 3 | 35785898 | 35786122 | mir-128-2 |
| 3 | 38010855 | 38011146 | mir-26a1 |
| 3 | 44155621 | 44155897 | mir-138-1 |
| 3 | 47890860 | 47891156 | mir-1226 |
| 3 | 49057422 | 49057699 | mir-425 |
| 3 | 49058003 | 49058247 | mir-191 |
| 3 | 52302264 | 52302428 | mir-let7g |
| 3 | 160122414 | 160122764 | mir-16-2 |
| 3 | 168269605 | 168269840 | mir-551b |
| 3 | 183959128 | 183959300 | mir-1224 |
| 4 | 8006885 | 8007215 | mir-95 |
| 4 | 20529837 | 20530095 | mir-218-1 |
| 4 | 38869601 | 38869810 | mir-574 |
| 4 | 115577880 | 115578166 | mir-577 |
| 5 | 58999365 | 58999658 | mir-582 |
| 5 | 87962556 | 87962837 | mir-9-2 |
| 5 | 136983185 | 136983462 | mir-874 |
| 5 | 148441822 | 148442076 | mir-584 |
| 5 | 148808354 | 148808642 | mir-143 |
| 5 | 148810152 | 148810472 | mir-145 |
| 5 | 149112238 | 149112561 | mir-378a |
| 5 | 159912286 | 159912506 | mir-146a |
| 5 | 167987818 | 167988126 | mir-103a-1 |
| 5 | 168195022 | 168195286 | mir-218-2 |
| 5 | 179225255 | 179225544 | mir-1229 |
| 6 | 52009069 | 52009263 | mir-206 |
| 6 | 72086439 | 72086784 | mir-30c-2 |
| 6 | 72113031 | 72113391 | mir-30a |
| 7 | 1062509 | 1062802 | mir-339 |
| 7 | 5535286 | 5535571 | mir-589 |
| 7 | 25989394 | 25989715 | mir-148a |
| 7 | 73605429 | 73605729 | mir-590 |
| 7 | 99691303 | 99691523 | mir-93 |
| 7 | 99691545 | 99691869 | mir-106b |
| 7 | 126697970 | 126698279 | mir-592 |
| 7 | 127847679 | 127848043 | mir-129-1 |
| 7 | 129410059 | 129410382 | mir-182 |
| 7 | 129414721 | 129415033 | mir-183 |
| 7 | 130561483 | 130561630 | mir-29a |
| 7 | 130562147 | 130562459 | mir-29b-1 |
| 7 | 136587866 | 136588177 | mir-490 |
| 7 | 150935470 | 150935795 | mir-671 |
| 7 | 157366973 | 157367151 | mir-153-2 |
| 8 | 9760866 | 9761202 | mir-124-1 |
| 8 | 14710920 | 14711249 | mir-383 |
| 8 | 22102363 | 22102696 | mir-320a |
| 8 | 41517910 | 41518235 | mir-486 |
| 8 | 65291616 | 65291947 | mir-124-2 |
| 8 | 135812635 | 135812951 | mir-30b |
| 8 | 135817085 | 135817276 | mir-30d |
| 8 | 141742611 | 141742895 | mir-151a |
| 9 | 4850259 | 4850569 | mir-101-2 |
| 9 | 20715934 | 20716260 | mir-491 |
| 9 | 28863576 | 28863837 | mir-876 |
| 9 | 28888718 | 28888995 | mir-873 |
| 9 | 73424746 | 73425019 | mir-204 |
| 9 | 86584631 | 86584848 | mir-7-1 |
| 9 | 96938205 | 96938411 | mir-let7a-1 |
| 9 | 96941092 | 96941292 | mir-let7d |
| 9 | 97847393 | 97847922 | mir-23b |
| 9 | 97848108 | 97848401 | mir-24-1 |
| 9 | 111808483 | 111808671 | mir-32 |
| 9 | 116971588 | 116971846 | mir-455 |
| 9 | 127454586 | 127454856 | mir-181a-2 |
| 9 | 127455788 | 127456129 | mir-181b-2 |
| 9 | 131154792 | 131155110 | mir-219-2 |
| 9 | 139564994 | 139565300 | mir-126 |
| 10 | 88024393 | 88024596 | mir-346 |
| 10 | 91352316 | 91352621 | mir-107 |
| 10 | 100154901 | 100155146 | mir-1287 |
| 10 | 104196136 | 104196413 | mir-146b |
| 10 | 105153956 | 105154184 | mir-1307 |
| 11 | 567935 | 568236 | mir-210 |
| 11 | 2155285 | 2155584 | mir-483 |
| 11 | 43602741 | 43603187 | mir-129-2 |
| 11 | 57408643 | 57408821 | mir-130a |
| 11 | 64658783 | 64659091 | mir-194-2 |
| 11 | 75045960 | 75046274 | mir-326 |
| 11 | 79113037 | 79113322 | mir-708 |
| 11 | 111384106 | 111384314 | mir-34c |
| 11 | 121970370 | 121970609 | mir-125b-1 |
| 11 | 122017030 | 122017391 | mir-let7a-2 |
| 11 | 122022884 | 122023222 | mir-100 |
| 12 | 7073051 | 7073378 | mir-200c |
| 12 | 54730877 | 54731193 | mir-148b |
| 12 | 58218281 | 58218587 | mir-26a-2 |
| 12 | 79812878 | 79813197 | mir-1252 |
| 12 | 81329477 | 81329793 | mir-618 |
| 12 | 95702108 | 95702351 | mir-331 |
| 12 | 97885495 | 97885778 | mir-1251 |
| 12 | 97957418 | 97957737 | mir-135a-2 |
| 13 | 92002689 | 92003002 | mir-20a |
| 13 | 92003231 | 92003804 | mir-92a-1 |
| 14 | 100575937 | 100576116 | mir-342 |
| 14 | 100774167 | 100774314 | mir-345 |
| 14 | 101318695 | 101318999 | mir-770 |
| 14 | 101335358 | 101335607 | mir-493 |
| 14 | 101341239 | 101341489 | mir-665 |
| 14 | 101348140 | 101348424 | mir-433 |
| 14 | 101377404 | 101377595 | mir-370 |
| 14 | 101488316 | 101488562 | mir-379 |
| 14 | 101489636 | 101489876 | mir-411 |
| 14 | 101490057 | 101490223 | mir-299 |
| 14 | 101491253 | 101491440 | mir-380 |
| 14 | 101492043 | 101492236 | mir-323a |
| 14 | 101492272 | 101492548 | mir-758 |
| 14 | 101493029 | 101493251 | mir-329-1 |
| 14 | 101493387 | 101493689 | mir-329-2 |
| 14 | 101495927 | 101496216 | mir-494 |
| 14 | 101498153 | 101498438 | mir-543 |
| 14 | 101499972 | 101500284 | mir-495 |
| 14 | 101506000 | 101506208 | mir-376c |
| 14 | 101506398 | 101506710 | mir-654 |
| 14 | 101506953 | 101507222 | mir-376a-1 |
| 14 | 101509222 | 101509537 | mir-1185-1 |
| 14 | 101510375 | 101510681 | mir-1185-2 |
| 14 | 101512110 | 101512405 | mir-381 |
| 14 | 101513618 | 101513942 | mir-539 |
| 14 | 101514121 | 101514414 | mir-889 |
| 14 | 101515750 | 101516014 | mir-655 |
| 14 | 101518728 | 101519012 | mir-487a |
| 14 | 101520556 | 101520858 | mir-382 |
| 14 | 101520951 | 101521179 | mir-134 |
| 14 | 101521688 | 101521869 | mir-485 |
| 14 | 101522470 | 101522694 | mir-323b |
| 14 | 101526054 | 101526220 | mir-154 |
| 14 | 101526855 | 101527171 | mir-496 |
| 14 | 101528364 | 101528541 | mir-377 |
| 14 | 101530700 | 101530991 | mir-541 |
| 14 | 101532156 | 101532371 | mir-410 |
| 14 | 101532861 | 101533160 | mir-656 |
| 14 | 102026486 | 102026778 | mir-1247 |
| 15 | 55665070 | 55665325 | mir-628 |
| 15 | 63116057 | 63116308 | mir-190a |
| 15 | 70371690 | 70371961 | mir-629 |
| 15 | 79502085 | 79502417 | mir-184 |
| 15 | 89151141 | 89151454 | mir-1179 |
| 15 | 89154927 | 89155201 | mir-7-2 |
| 15 | 89911212 | 89911397 | mir-9-3 |
| 16 | 2321709 | 2321981 | mir-940 |
| 16 | 14397740 | 14397935 | mir-193b |
| 16 | 14403004 | 14403332 | mir-365a |
| 16 | 15737054 | 15737365 | mir-484 |
| 16 | 56892325 | 56892593 | mir-138-2 |
| 16 | 67236054 | 67236322 | mir-328 |
| 16 | 69966843 | 69967161 | mir-140 |
| 17 | 1953169 | 1953327 | mir-132 |
| 17 | 1953469 | 1953699 | mir-212 |
| 17 | 6921170 | 6921445 | mir-497 |
| 17 | 7126537 | 7126866 | mir-324 |
| 17 | 11985135 | 11985387 | mir-744 |
| 17 | 19247639 | 19247932 | mir-1180 |
| 17 | 28443992 | 28444293 | mir-423 |
| 17 | 29886931 | 29887182 | mir-193a |
| 17 | 46114483 | 46114727 | mir-152 |
| 17 | 46657137 | 46657406 | mir-10a |
| 17 | 56408525 | 56408719 | mir-142 |
| 17 | 57228432 | 57228734 | mir-301a |
| 17 | 57918470 | 57918717 | mir-21 |
| 17 | 79099456 | 79099789 | mir-338 |
| 17 | 79106935 | 79107224 | mir-1250 |
| 18 | 19263348 | 19263671 | mir-320c-1 |
| 18 | 19405490 | 19405799 | mir-133a-1 |
| 18 | 33484731 | 33485049 | mir-187 |
| 18 | 56118099 | 56118425 | mir-122 |
| 19 | 4770603 | 4770930 | mir-7-3 |
| 19 | 10928012 | 10928263 | mir-199a-1 |
| 19 | 13947151 | 13947395 | mir-27a |
| 19 | 13985490 | 13985912 | mir-181d |
| 19 | 40788427 | 40788754 | mir-641 |
| 19 | 46142112 | 46142465 | mir-330 |
| 19 | 46522128 | 46522352 | mir-769 |
| 19 | 50003981 | 50004241 | mir-150 |
| 19 | 52195912 | 52196229 | mir-99b |
| 19 | 52196245 | 52196612 | mir-let7e |
| 19 | 54240043 | 54240249 | mir-516b-1 |
| 19 | 54485414 | 54485719 | mir-935 |
| 20 | 3898083 | 3898256 | mir-103a2 |
| 20 | 26188796 | 26188939 | mir-663a |
| 20 | 33578118 | 33578359 | mir-499a |
| 20 | 57392647 | 57392794 | mir-296 |
| 20 | 61151446 | 61151750 | mir-1-1 |
| 20 | 61162071 | 61162375 | mir-133a-2 |
| 20 | 61809767 | 61810041 | mir-124-3 |
| 21 | 17911300 | 17911507 | mir-99a |
| 21 | 17912079 | 17912324 | mir-let7c |
| 21 | 17962359 | 17962665 | mir-125b2 |
| 21 | 26946267 | 26946586 | mir-155 |
| 22 | 20020569 | 20020819 | mir-185 |
| 22 | 20236615 | 20236857 | mir-1286 |
| 22 | 22007134 | 22007429 | mir-301b |
| 22 | 22007549 | 22007827 | mir-130b |
| 22 | 38243640 | 38243806 | mir-659 |
| X | 8094904 | 8095211 | mir-651 |
| X | 45605463 | 45605798 | mir-221 |
| X | 45606244 | 45606603 | mir-222 |
| X | 49767730 | 49767933 | mir-532 |
| X | 49773017 | 49773317 | mir-500a |
| X | 49773527 | 49773819 | mir-362 |
| X | 49774274 | 49774449 | mir-501 |
| X | 49775177 | 49775377 | mir-500b |
| X | 49779120 | 49779355 | mir-502 |
| X | 53583086 | 53583369 | mir-98 |
| X | 53584123 | 53584258 | let-7f-2 |
| X | 63005816 | 63005991 | mir-1468 |
| X | 65238658 | 65238843 | mir-223 |
| X | 73438168 | 73438479 | mir-421 |
| X | 85158606 | 85158834 | mir-361 |
| X | 109298439 | 109298755 | mir-652 |
| X | 113949575 | 113949841 | mir-1298 |
| X | 114057975 | 114058181 | mir-448 |
| X | 117520246 | 117520569 | mir-1277 |
| X | 118780677 | 118780854 | mir-766 |
| X | 133303484 | 133303662 | mir-92a-2 |
| X | 133675317 | 133675648 | mir-542 |
| X | 133680164 | 133680447 | mir-503 |
| X | 133680590 | 133680914 | mir-424 |
| X | 137749837 | 137749985 | mir-504 |
| X | 139006222 | 139006409 | mir-505 |
| X | 140008219 | 140008486 | mir-320d-2 |
| X | 146318359 | 146318565 | mir-508 |
| X | 146341134 | 146341320 | mir-509-3 |
| X | 146342001 | 146342267 | mir-509-1 |
| X | 151127000 | 151127303 | mir-224 |
| X | 151127929 | 151128206 | mir-452 |
| X | 151560610 | 151560808 | mir-105-1 |
| X | 151561723 | 151562024 | mir-767 |
| X | 151562806 | 151563090 | mir-105-2 |

Footnote: ^a^According to the human reference sequence - Human Build 37/human genome 19; ^b^miRNA nomenclature according to mirbase 22.1 (<https://www.mirbase.org/>)

**B**


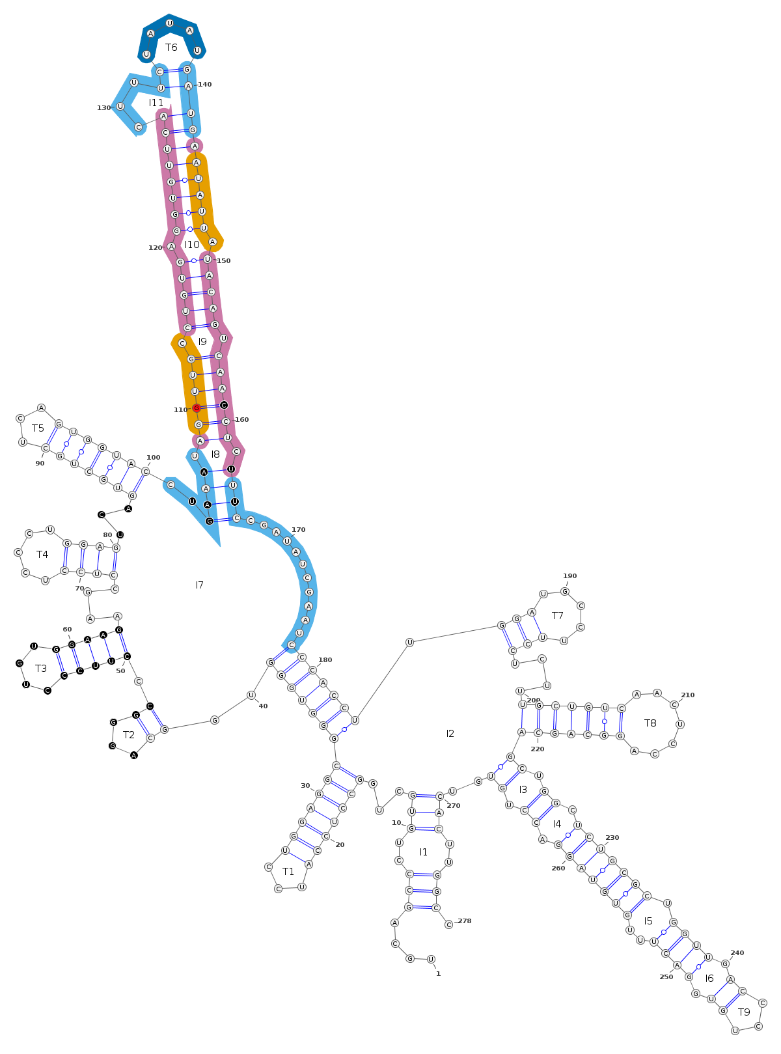

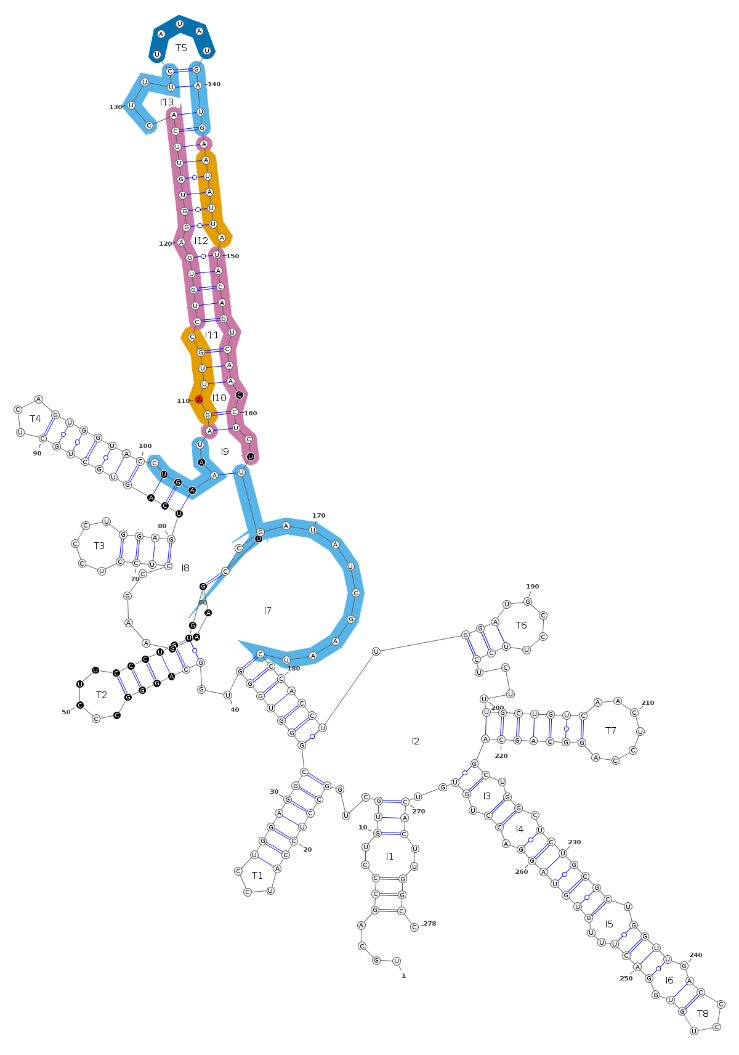


**A**

**S1 Figure: Impact of *MIR656* seed variant on miRNA structure.** Maximal expected accuracy is shown for the A) reference and the B) alternative allele. Structural changes occur in both 5p and 3p arms of the lower stem. We also notice that the presence of the variant creates a small loop. Red dot indicates the variant location. Nucleotides with altered base pairing are shown in black.
